# Nonstoichiometric Defect Engineering in Mn^2+^‐Doped MgGa_2_O_4_ Persistent Phosphors Toward Enhanced Luminescence and Multimodal Optical Manometry

**DOI:** 10.1002/advs.78629

**Published:** 2026-11-03

**Authors:** Peng Du, Yifei Yu, Yue Guo, Przemysław Woźny, Shualing Ma, Laihui Luo, Marcin Runowski

**Affiliations:** ^1^ School of Physical Science and Technology Ningbo University Ningbo Zhejiang China; ^2^ School of Applied Physics and Materials Wuyi University Jiangmen Guangdong China; ^3^ Faculty of Chemistry Adam Mickiewicz University Poznań Poland

**Keywords:** afterglow emission, high‐pressure experiments, luminescent pressure sensors, Mn^2+^ luminescence, optical manometry

## Abstract

Mn^2+^‐doped MgGa_2_O_4_ persistent phosphors were synthesized through a nonstoichiometric engineering strategy to precisely regulate their luminescence properties. By introducing Mg^2+^ deficiency, the luminescence intensity, thermal stability and persistent luminescence performance are simultaneously enhanced, enabling their potential application in optical anti‐counterfeiting. Furthermore, the in situ high‐pressure X‐ray diffraction measurements demonstrate the excellent structural robustness of the designed phosphors over the investigated pressure range, indicating their suitability for high‐pressure manometry. Upon compression, the resultant phosphors exhibit pronounced pressure‐induced spectral red‐shift, enabling tunable luminescence and visualized pressure sensing. Notably, compared with the stoichiometric counterpart, the Mg^2+^‐deficient phosphors display significantly enhanced pressure responsiveness, resulting in improved optical manometric performance with higher sensitivity and an extended working pressure range. Specifically, within a broad pressure range of 0–30 GPa, the Mg_0.939_Ga_2_O_4_:Mn^2+^ phosphor achieve pressure sensitivities of 5.01 and 2.29 nm GPa^−1^ based on the emission band centroid and full width at half maximum, respectively. Additionally, the developed phosphors exhibit good colorimetric pressure sensing capability, reaching a maximum relative sensitivity of 12.71% GPa^−1^. These results demonstrate that the optical properties of the Mn^2+^‐doped MgGa_2_O_4_ phosphors can be effectively modulated through the synergistic regulation of nonstoichiometric engineering and pressure, highlighting their application potential in optical anti‐counterfeiting and multimodal manometry.

## Graphical abstract

Mg^2+^ deficiency transforms Mn^2+^‐doped MgGa_2_O_4_ into a multifunctional persistent phosphor, enhancing green emission, thermal stability, and afterglow effect for optical anti‐counterfeiting. Under compression, spectral shifts enable visual pressure sensing with a sensitivity of 5.01 nm GPa^−1^ over a pressure range up to 30 GPa. Defect engineering thus offers a promising route to high‐performance optical manometry.

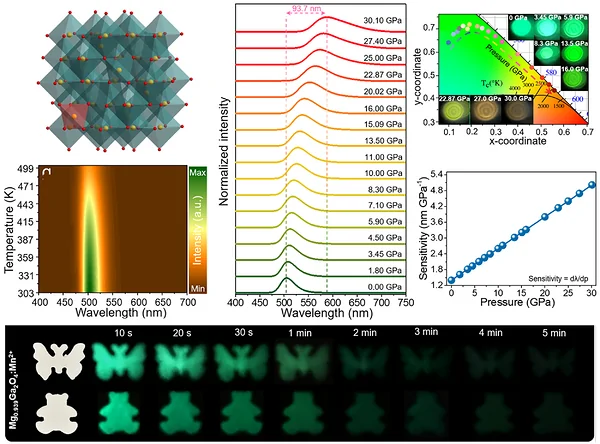

## 1 Introduction

Luminescent materials activated by rare‐earth or metal ions, which are capable of converting excitation light into desirable emissions spanning from ultraviolet to infrared radiation, have attracted extensive attention due to their broad applications in solid‐state lighting, optical sensing, anti‐counterfeiting, X‐ray imaging, and other advanced photonic technologies [1, 2, 3, 4, 5]. Recently, luminescent materials doped with different d‐block metal ions (i.e., Fe^3+^, Mn^2+^, Cr^3+^, Bi^3+^, Cu^2+^, etc.) have received increasing research interest owing to their distinctive advantages, including low cost, eco‐friendly feature, facile synthesis and versatile emission characteristics [6, 7, 8, 9, 10]. Among them, phosphors doped with Mn^2+^, which exhibit the electronic configuration of [Ar]3d^5^4s^2^, attract special attention, mainly due to their controllable optical properties [11, 12]. As is well documented, the luminescence of Mn^2+^ results from d–d transition, of which the 3d shell is half‐filled by five d‐electrons, and its emission band spectral position and intensity are largely impacted by the surrounding crystal field [13, 14]. Thus, precisely manipulating the local crystal field surrounding Mn^2+^ presents an effective strategy for tailoring its luminescence performance. To date, numerous approaches, such as host selection, heterovalent substitution, defect engineering, compositional regulation, etc., have been proposed to modulate the optical properties of Mn^2+^‐doped phosphors [15, 16, 17]. Specifically, nonstoichiometric engineering has recently emerged as a powerful approach for optimizing luminescence properties because it enables flexible regulation of lattice composition, defect structures, charge compensation mechanisms, and radiative transition probabilities. Using nonstoichiometric engineering, Song et al. revealed that the valence state of manganese ions in Ba_0.75_Al_11_O_17.25_ lattice was regulated, leading to controllable luminescence and multifunctional optical applications [18]. Kim et al. reported that excessive incorporation of precursor components induced a phase transition in Mn^2+^‐doped ZnSiO_4_ phosphors, resulting in tunable luminescence from green to yellow [19]. Inspired by these advances, further exploration of nonstoichiometric strategies for regulating Mn^2+^ luminescence and developing high‐performance multifunctional optical materials is highly desirable.

To meet the requirements of practical applications, the designed luminescent materials should highly efficient luminescence characteristics, of which the selection of a proper host is regarded as a facile way to realize this goal. Nowadays, spinel‐type oxides with the general chemical formula of AB_2_O_4_ (A and B refers to divalent and trivalent metal ions, respectively) have attracted considerable attention as promising host matrices for metal ions [20, 21]. Zhong et al. developed the Bi^3+^‐doped MgGa_2_O_4_ phosphors with multimodal emissions, demonstrating their potential for advanced optical anti‐counterfeiting applications [22]. By introducing Mn^2+^ into MgAlGaO_4_ lattice, controllable luminescence ranging from the visible to near‐infrared region was achieved, enabling multifunctional lighting applications [23]. Qiu et al. stated that the Cr^3+^‐doped MgAl_2_O_4_ phosphors with intense near‐infrared emission were suitable for light‐emitting diode [24]. Notably, most previous studies have focused on the luminescence properties of metal‐ion‐doped spinel‐type compounds under ambient conditions, whereas the evolution of their optical properties in response to compositional regulation (e.g., nonstoichiometric engineering) and extreme external environments (e.g., high pressure) remains largely unexplored. As is well known, high pressure, as an effective external stimulus, can continuously compress the lattice, shorten interatomic distances, and modify the crystal‐field strength and metal–ligand covalency, thereby providing a unique means of tuning the luminescence characteristics of luminescent centers [25, 26]. Moreover, by establishing quantitative correlations between pressure and spectral parameters, i.e., emission peak position, bandwidth, fluorescence lifetime, and fluorescence intensity ratio, etc., optical pressure sensing can be realized with high spatial resolution and remote detection capability [27, 28, 29]. Furthermore, some Mn^2+^‐doped phosphors, such as MgAl_2_O_4_:Mn^2+^, Zn_2_GeO_4_:Mn^2+^, NaY_9_(SiO_4_)_6_O_2_:Mn^2+^, Lu_2_Mg_2_Al_2_Si_2_O_12_:Mn^2+^, Mg_2_Y_8_(SiO_4_)_6_O_2_:Mn^2+^, Ca_2_Gd_8_(SiO_4_)_6_O_2_:Mn^2+^, etc. [30, 31, 32, 33, 34, 35], had been demonstrated to exhibit pressure‐responsive luminescence behaviors, providing promising platforms for developing highly sensitive optical manometers. However, the influence of nonstoichiometric regulation on the pressure‐dependent luminescence response of Mn^2+^‐doped spinel‐type phosphors remains unclear. In particular, the synergistic relationship between defect/composition modulation and pressure‐induced luminescence evolution has not yet been systematically established. Therefore, several fundamental questions remain to be addressed: (i) how does nonstoichiometric engineering affect the pressure‐induced spectral evolution of Mn^2+^‐doped spinel‐type phosphors, and (ii) can nonstoichiometric regulation further enhance the pressure sensitivity and sensing capability compared with conventional stoichiometric compositions? Addressing these questions is essential for developing multifunctional luminescent materials with controllable emission properties and advanced optical sensing capabilities.

In this work, we first identified the optimal Mn^2+^ doping concentration as a baseline, and then systematically explored how Mg^2+^ deficiency modulated the luminescence behaviors of Mn^2+^ in MgGa_2_O_4_ lattices. Herein, the Mg_1‐_ *_x_* Ga_2_O_4_:*x*Mn^2+^ (MgGa_2_O_4_:*x*Mn^2+^) and Mg_0.999‐_ *_y_* Ga_2_O_4_:0.001Mn^2+^ (Mg_0.999_ *_‐y_* Ga_2_O_4_:Mn^2+^) persistent phosphors were rationally designed and synthesized. Under UV excitation, the obtained phosphors exhibit intense green emission originating from the d‐d transition of Mn^2+^. By employing a nonstoichiometric engineering strategy through Mg^2+^ deficiency regulation, the luminescence intensity, thermal stability and persistent (afterglow) luminescence performance were simultaneously improved, enabling their potentials for optical anti‐counterfeiting. Moreover, the structural stability and lattice evolution of the optimized phosphors under external compression were systematically investigated by in situ high‐pressure X‐ray diffraction (XRD) characterization. Upon applying pressure, the synthesized phosphors exhibit remarkable pressure‐induced polychromatic luminescence, characterized by continuously tunable emission colors, providing a visual approach for pressure sensing. Notably, the Mg^2+^‐deficient phosphors demonstrate enhanced pressure sensitivity compared with their stoichiometric counterparts, resulting in superior optical manometric performance. These findings revealed that nonstoichiometric engineering is an effective strategy for simultaneously optimizing persistent luminescence and pressure‐responsive optical behaviors, and highlight Mg_0.939_Ga_2_O_4_:Mn^2+^ phosphors as promising multifunctional compounds for optical anti‐counterfeiting and high‐performance pressure sensing.

## 2 Experimental Section

### 2.1 Synthesis of the Mn^2+^‐Doped MgGa_2_O_4_ Persistent Phosphors

The Mg_1‐_ *_x_* Ga_2_O_4_:*x*Mn^2+^ (MgGa_2_O_4_:*x*Mn^2+^; 0.0005 ≤ *x* ≤ 0.011) and Mg_0.999‐_ *_y_* Ga_2_O_4_:0.001Mn^2+^ (Mg_0.999_ *_‐y_* Ga_2_O_4_:Mn^2+^; 0.00 ≤ *y* ≤ 0.10) persistent phosphors were synthesized via a high‐temperature solid‐state reaction method. The MgO, Ga_2_O_3_ and MnCO_3_ powders were adopted as the raw materials. For preparation of the MgGa_2_O_4_:*x*Mn^2+^ phosphors, stoichiometric amounts of the raw materials were accurately weighted, mixed thoroughly, and subsequently transferred into alumina crucibles. The mixed powders were sintered at 1400°C for 6 h. After naturally cooling to room temperature, the obtained products were reground and collected for subsequent structural and optical characterizations. As for the synthesis of the Mg^2+^‐deficient compounds, i.e., Mg_0.999_ *_‐y_* Ga_2_O_4_:Mn^2+^, the same synthetic procedure and calcination conditions were employed, except that the MgO amount was precisely reduced according to the designed nonstoichiometric compositions.

### 2.2 Characterization

The phase structures of the synthesized products were identified by X‐ray diffractometer (Bruker D8 Advance) equipped with Cu K*α* radiation (*λ* = 1.5406 Å), while the X‐ray photoelectron spectroscopy (XPS) measurement was carried out by a Thermo Scientific K‐*α* spectrometer. Using an electron paramagnetic response (EPR) spectrometer (Bruker EMXplus‐6/1), the EPR spectrum of the representative phosphor was tested. The luminescence properties, i.e., emission and excitation spectra, of the designed compounds were recorded through a fluorescence spectrometer (Edinburgh FS5). Moreover, with the help of a field‐emission scanning microscopy (FE‐SEM; HITACHI SU3500) and transmission electron microscopy (TEM; FEI Tecnai F20), the structure and morphology of the resulting phosphors were investigated. The luminescence decay curves of the targeted materials were examined by means of a fluorescence spectrometer (ZoLix Omni‐λ3005i). The thermoluminescence (TL) spectra of the samples were recorded using an SL18 TL measurement system, with a 254 nm UV lamp (6 W) employed as the excitation source.

High‐pressure experiments were conducted using a symmetric diamond anvil cell (DAC), with the pressure calibrated by the fluorescence shift of a ruby pressure sensor. A small amount of prepared phosphors was loaded into a sample chamber (i.e., diameter of ≈150 µm) within a stainless‐steel gasket with an initial thickness of 250 µm. During the high‐pressure measurement, the mixture of methanol/ethanol/water (i.e., volume ratio of 16:3:1) was employed as the pressure‐transmitting medium. The in‐situ high‐pressure emission spectra were collected under 266 nm excitation using an Andor Shamrock 500i spectrometer equipped with a silicon CCD detector. Furthermore, in‐situ high‐pressure XRD patterns were conducted at BL17U1 beamline of Shanghai Synchrotron Radiation Facility (SSRF), in which the pressure inside the DAC chamber was measured by the equation of state of Au.

## 3 Results and Discussion

The crystal structure of MgGa_2_O_4_ unit cell is illustrated in Figure 1b. As observed, Mg and Ga cations occupy the same crystallographic sites, i.e., they can both reside at two distinct types of sites. Specifically, Mg and Ga cations can simultaneously occupy tetrahedral and octahedral sites, resulting in the formation of intrinsic anti‐site defects [36]. In the MgGa_2_O_4_ lattice, the Mg/GaO_4_ tetrahedra are connected with Mg/GaO_6_ octahedra through corner sharing, whereas adjacent Mg/GaO_6_ octahedra are linked via edge sharing. When Mn^2+^ is introduced into the host lattice, it is expected to preferentially occupy the tetrahedral Mg^2+^ site due to their comparable ionic radii and coordination preference [37], resulting in the MnO_4_ tetrahedron, as shown in Figure 1b. To explore the influence on the Mn^2+^ incorporation on the phase structure of the studied samples, the powder XRD data for the MgGa_2_O_4_:*x*Mn^2+^ phosphors were collected and presented in Figure 1c. Apparently, all diffraction peaks remain unchanged with increasing Mn^2+^ concentration, and they can be well indexed to the standard cubic MgGa_2_O_4_ (PDF#10‐0113), indicating that Mn^2+^ doping does not induce secondary phase formation and that all products retain a stable single‐phase cubic structure. In general, the formation of a substitutional solid solution requires a relatively small radii difference (*D*_R_) between the ionic radii of the dopant and substituted ions, which can be evaluated using the equation of *D*_R_ = |*R*_r_ − *R*_d_|/*R*_r_  × 100% (where *R*_r_ and *R*_d_ represent the ionic radii of replaced and doping ions, respectively) [15, 30]. Consequently, the *D*_R_ value between Mg^2+^ (i.e., *R_r_* = 0.57 Å) and Mn^2+^ (i.e., *R*_d_ = 0.66 Å) is decided to be 15.8%, which is significantly smaller than the empirical criterion of 30%, revealing that the Mn^2+^ can replace the Mg^2+^ position to form the MgGa_2_O_4_:*x*Mn^2+^ phosphors. Meanwhile, the influence of Mg^2+^ deficiency on the phase stability of the resulting phosphors was investigated. As shown in Figure 1d, no obvious changes in diffraction peak positions or additional impurity phases are observed with increasing Mg^2+^ deficiency, demonstrating that the MgGa_2_O_4_ host structure remains stable over the entire investigated nonstoichiometric composition range. For the purpose of obtaining deeper insights into the crystal structures of the designed phosphors, the Rietveld refinements were performed based on the XRD patterns of the MgGa_2_O_4_:0.001Mn^2+^ and Mg_0.939_Ga_2_O_4_:Mn^2+^ phosphors, with the corresponding results displayed in Figure 1e,f, respectively. The calculated diffraction profiles exhibit excellent agreement with the experimental data, further confirming the formation of a single cubic phase of the resultant phosphors. Furthermore, the Rietveld XRD refinements shown in Figure S1 reveal that the other Mg^2+^‐deficient compounds, i.e., Mg_0.979_Ga_2_O_4_:Mn^2+^, Mg_0.959_Ga_2_O_4_:Mn^2+^, Mg_0.919_Ga_2_O_4_:Mn^2+^ and Mg_0.899_Ga_2_O_4_:Mn^2+^, also possess pure cubic phase, further manifesting that the utilization of Mg^2+^ deficiency engineering cannot alter the phase structure of the as‐prepared samples. Additionally, the refined lattice parameters gradually decrease with increasing Mg^2+^ deficiency (Table S1), indicating lattice contraction induced by nonstoichiometric regulation. Such lattice shrinkage is expected to modify the local crystal‐field environment surrounding Mn^2+^, thereby affecting the luminescence behavior, as discussed in the following section.

**FIGURE 1:**
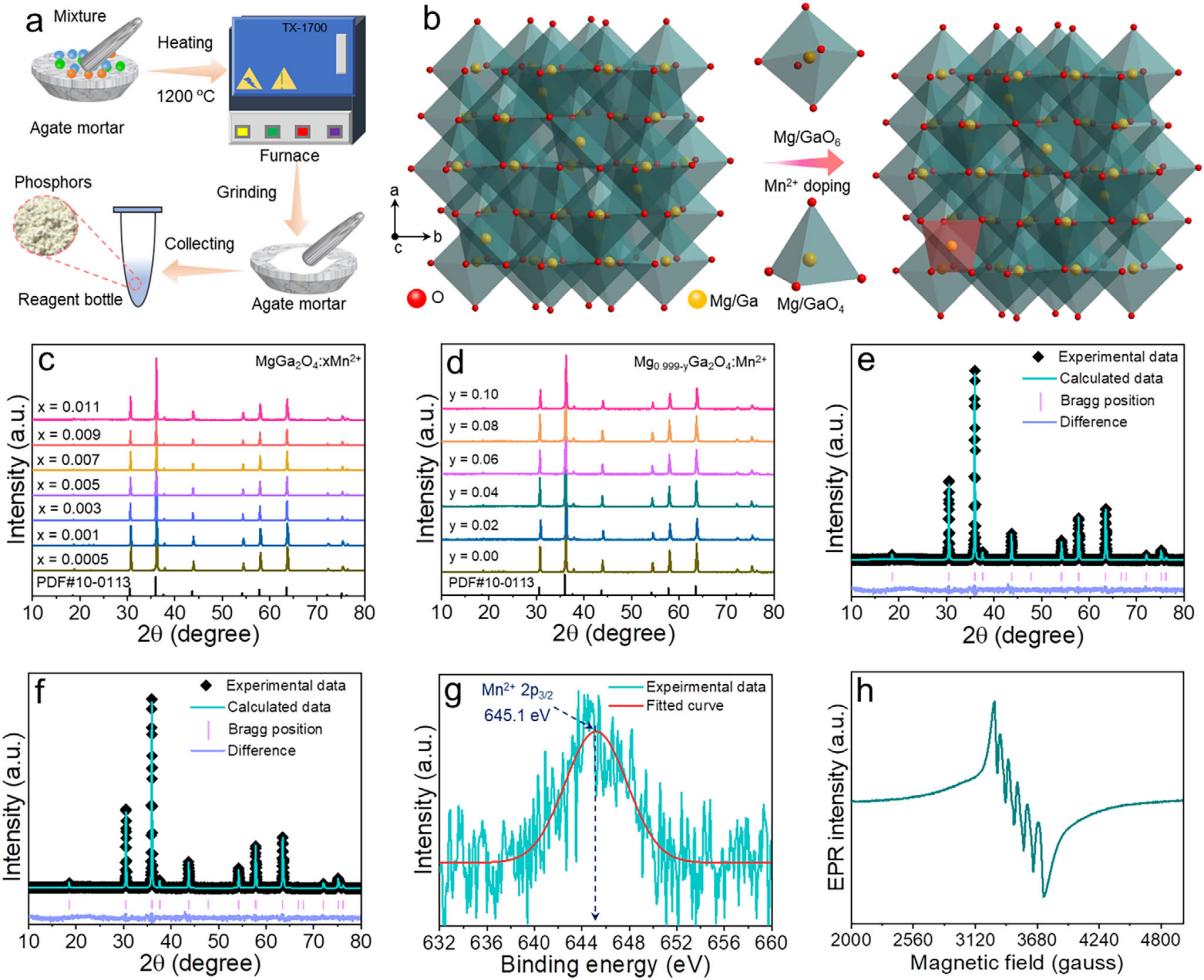
(a) Schematic diagram for the preparation process of the designed phosphors. (b) 3D representations of crystal structures for the MgGa_2_O_4_ (left) and Mn^2+^‐doped MgGa_2_O_4_ (right) phosphors. Powder XRD patterns of the (c) MgGa_2_O_4_:*x*Mn^2+^ and (d) Mg_0.999‐_ *_y_* Ga_2_O_4_:Mn^2+^ phosphors. Rietveld XRD refinements of the optimized (e) MgGa_2_O_4_:0.001Mn^2+^ and (f) Mg_0.939_Ga_2_O_4_:Mn^2+^ phosphors. (g) High‐resolution XPS spectrum for Mn^2+^ 2p and (h) EPR spectrum of the MgGa_2_O_4_:0.001Mn^2+^ phosphor.

XPS measurement was conducted to determine the elemental composition and chemical states of the constituent elements in the optimized phosphor MgGa_2_O_4_:0.001Mn^2+^. The full‐survey XPS spectrum, shown in Figure S1a, confirms the presence of Mg, Ga, O and Mn in the studied sample. The high‐resolution XPS spectrum of Mg 2p (Figure S1b) is dominated by an intense band at around 1303.7 eV corresponding to the Mg 2p_3/2_ [34], while that of the Ga 2p (Figure S1c) contains two sharp peaks at about 1117.9 and 1144.8 eV, which are attributed to the Ga 2p_3/2_ and Ga 2p_1/2_, respectively [38]. Whereas, the high‐resolution XPS spectrum of O 1s (Figure S1d) reveals a strong, single band at 530.1 eV. Finally, the successful incorporation and valence state of manganese ions were further verified by the high‐resolution XPS spectrum, of which a single band with the binding energy of 645.1 eV (i.e., Mn 2p_3/2_) is observed in Figure 1g [34, 35]. To further confirm the valance state of manganese ions in the designed compounds, the EPR analysis of the optimized MgGa_2_O_4_:0.001Mn^2+^ phosphor was performed and the corresponding result is illustrated in Figure 1h. Significantly, the recorded EPR spectrum exhibits six well‐resolved hyperfine splitting signals, originating from the interaction between the electron spin of Mn^2+^ (*S* = 5/2) and its nuclear spin (*I* = 5/2), suggesting that divalent manganese dopant ions are present in the MgGa_2_O_4_ host lattice [15, 30]. Additionally, the calculated effective *g* value of approximately 2.03 suggests that Mn^2+^ occupies a tetrahedrally coordinated environment [15, 30], which is in good agreement with the discussion based on the crystal structure.

The morphology evolution of the developed phosphors was characterized by SEM and TEM techniques. As shown in the SEM image (Figure 2a), the MgGa_2_O_4_:*x*Mn^2+^ phosphor consists of irregularly shaped microparticles, which can be attributed to the high‐temperature solid‐state reaction process. With increasing Mn^2+^ concentration, no obvious changes in particle morphology, including particle shape and size distribution, are observed (see Figure S3), indicating that Mn^2+^ incorporation has a negligible influence on the materials morphology. Furthermore, the introduction of Mg^2+^ deficiency also does not induce noticeable morphological variation, as demonstrated by the SEM images of Mg_0.939_Ga_2_O_4_:Mn^2+^ and Mg_0.899_Ga_2_O_4_:Mn^2+^ samples (Figure 2b,c). These results suggest that the designed nonstoichiometric regulation strategy can only modify the lattice composition without altering the overall particle morphology. The TEM images further confirm that the prepared phosphors are composed of irregular microparticles (Figure 2d and Figure S4a). The corresponding high‐resolution TEM images display well‐resolved lattice fringes (Figure 2e and Figure S4b), with an interplanar spacing of approximately 2.50 Å, which can be assigned to the (311) plane of cubic MgGa_2_O_4_ (PDF#10‐0113). Moreover, the selected‐area electron diffraction (SAED) pattern of the Mg_0.939_Ga_2_O_4_:Mn^2+^ phosphor exhibits distinct diffraction spots (Figure 2f), confirming high crystallinity of the synthesized phosphors. Similar diffraction features are also observed for the Mg_0.899_Ga_2_O_4_:Mn^2+^ sample (Figure S4c). Additionally, energy‐dispersive X‐ray spectroscopy (EDS) analysis also confirms the presence of Mg, Ga, O and Mn elements in the prepared phosphors (Figure 2g and Figure S4d). The corresponding elemental mapping images (Figure 2h–k and Figure S4e–h) reveal that these elements are homogeneously distributed throughout the particles, demonstrating the successful incorporation and uniform dispersion of Mn^2+^ within the MgGa_2_O_4_ lattice. Furthermore, on the basis of the EDS results, it is clear that the Mg/Ga ratios in the resulting phosphors decrease gradually as Mg^2+^ deficiency increases, which coincides well with the precursor design (Table S2; see discussion in the Supporting Information file for details). These results demonstrate that the utilization of Mg^2+^ deficiency is an effective strategy to modulate the Mg/Ga ratio in the final products, leading to the modified luminescence features.

**FIGURE 2:**
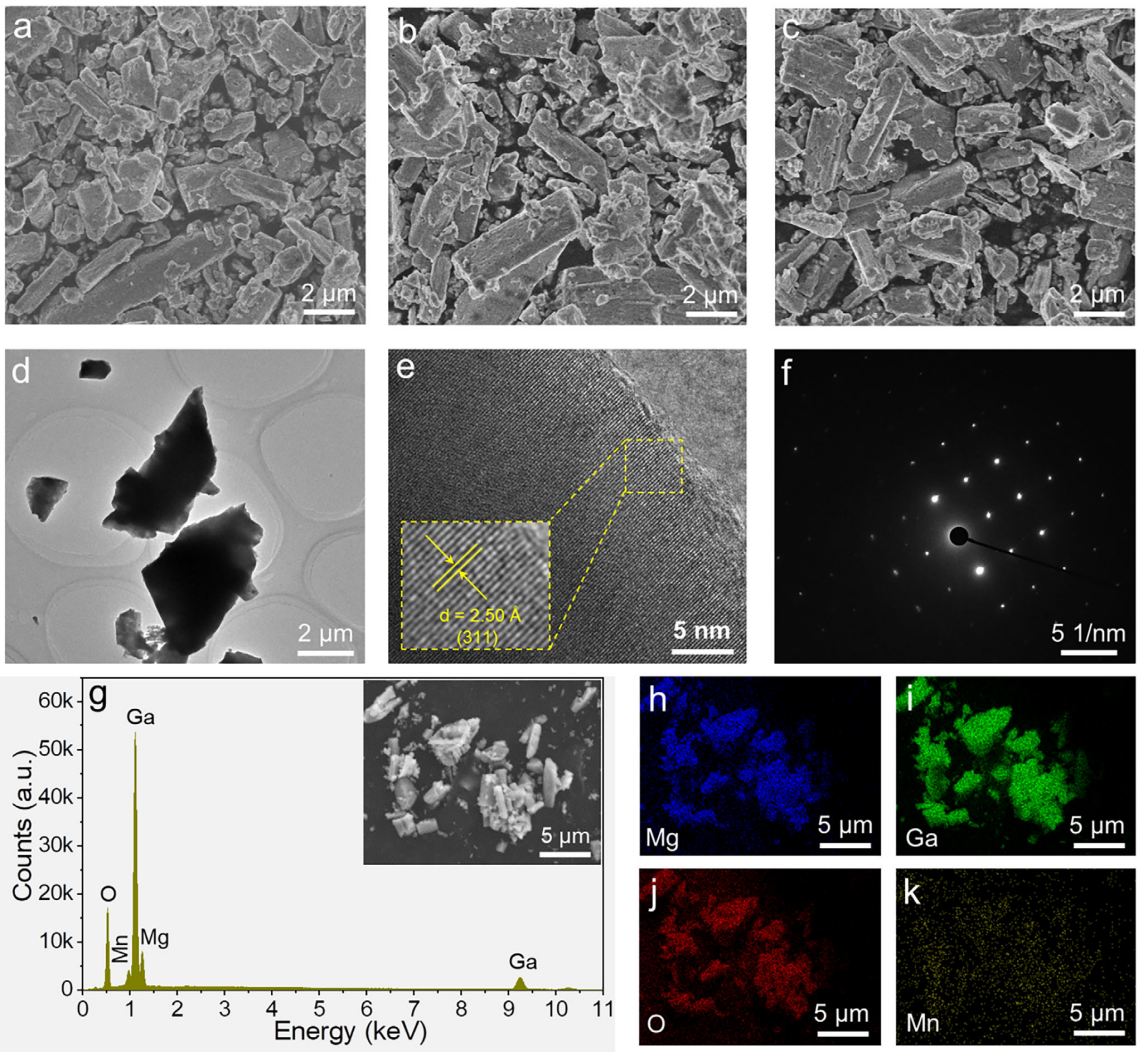
SEM images of the (a) MgGa_2_O_4_:0.001Mn^2+^, (b) Mg_0.939_Ga_2_O_4_:Mn^2+^ and (c) Mg_0.899_Ga_2_O_4_:Mn^2+^ phosphors. (d) TEM image, (e) High‐resolution TEM image, (f) SAED pattern, (g) EDS spectrum and (h‐k) Elemental mapping results for the Mg_0.939_Ga_2_O_4_:Mn^2+^ phosphor.

### 3.1 Luminescence Studies

Figure 3a presents the excitation and emission spectra of the MgGa_2_O_4_:0.001Mn^2+^ phosphor. The excitation spectrum monitored at 504 nm consists of two bands at approximately 227 and 270 nm, which can be assigned to the host absorption and O^2−^–Mn^2+^ charge transfer (CT) band, respectively [37, 39]. Upon excitation at 227 nm, an intense band centered at around 504 nm, corresponding to the ^4^T_1_(^4^G) → ^6^A_1_ transition of Mn^2+^, is observed [30, 31], suggesting that the Mn^2+^ takes up the tetrahedral site in the MgGa_2_O_4_ lattice, which is consistent with the structural analysis and EPR results. To determine the optimal Mn^2+^ doping concentration, the concentration‐dependent luminescence behaviors of the MgGa_2_O_4_:*x*Mn^2+^ phosphors were systematically characterized (Figure 3b). With increasing Mn^2+^ content, the emission intensity varies significantly without altering the shape of the spectral profile, and the optimal doping content is found to be *x* = 0.001 in the MgGa_2_O_4_:*x*Mn^2+^ compounds. Further increasing the Mn^2+^ concentration results in obvious concentration quenching. The FWHM value of the obtained emission in MgGa_2_O_4_:0.001Mn^2+^ phosphor is 30.2 nm, which is almost unchanged as Mn^2+^ content raises (see Table S3). The calculated critical distance (i.e., *R*_c_ = 51.3 Å) suggests that the multipolar interaction contributes to the concentration quenching mechanism (see discussion in the Supporting Information file for details). Furthermore, by analyzing the relation between fluorescence intensity and doping content, the involved concentration quenching mechanism is further identified as an electric dipole‐dipole interaction (see Figure S5). Subsequently, the effect of nonstoichiometric engineering (i.e., Mg^2+^ deficiency) on the luminescence performance of the studied samples was explored by designing a series of the Mg_0.999_ *_‐y_* Ga_2_O_4_:Mn^2+^ phosphors. As shown in Figure 3c, Mg^2+^ deficiency does not induce any variation in the emission profile but significantly enhances the emission intensity. The luminescence enhancement can be attributed to the regulation of the local coordination environment and crystal‐field surrounding Mn^2+^ induced by Mg^2+^ deficiency. The maximum emission intensity is obtained at *y* = 0.06 (Figure 3c), demonstrating the effectiveness of Mg^2+^ deficiency engineering in optimizing the luminescence performance. Although the luminescence intensity is enhanced, the emission bandwidth is hardly changed by employing the Mg^2+^ deficiency engineering (Table S3). Additionally, the resulting compounds can emit bright green light upon UV light irradiation, with the color coordinates for the MgGa_2_O_4_:0.001Mn^2+^ and Mg_0.939_Ga_2_O_4_:Mn^2+^phosphors of (0.071,0.658) and (0.074,0.648), respectively (Figure 3d). These results indicate that the nonstoichiometric regulation strategy effectively enhances emission intensity while maintaining stable emission color.

**FIGURE 3:**
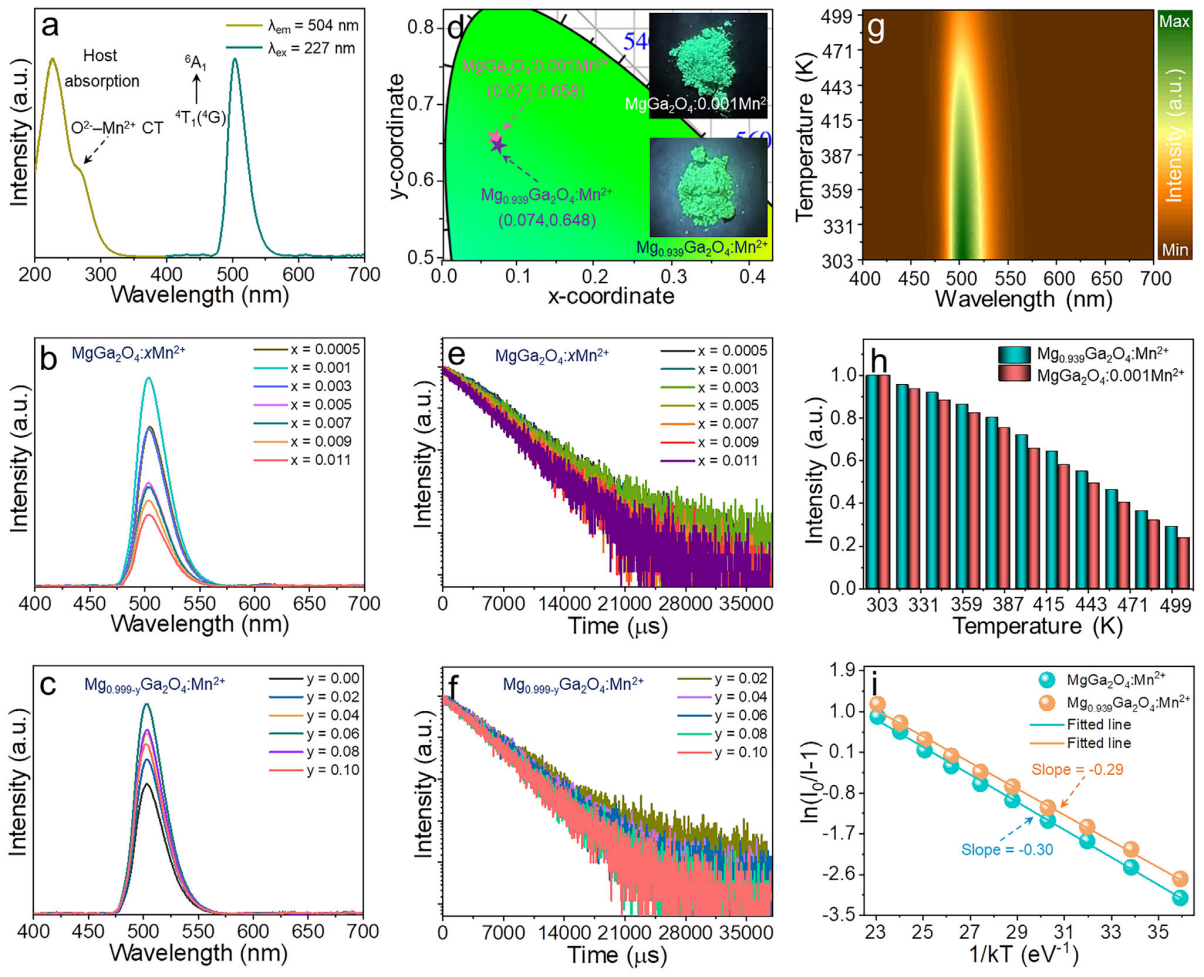
(a) Excitation and emission spectra of the MgGa_2_O_4_:0.001Mn^2+^ phosphor. Emission spectra of the (b) MgGa_2_O_4_:*x*Mn^2+^ and (c) Mg_0.999‐_ *_y_* Ga_2_O_4_:Mn^2+^ phosphors. (d) CIE chromaticity diagram of the resulting phosphors. Decay curves of the (e) MgGa_2_O_4_:*x*Mn^2+^ and (f) Mg_0.999‐_ *_y_* Ga_2_O_4_:Mn^2+^ phosphors. (g) Temperature‐dependent emission spectra of the Mg_0.939_Ga_2_O_4_:Mn^2+^ phosphor. (h) Temperature‐dependent fluorescence intensities and (i) Plots of ln(*I*_0_/*I*‐1) vs 1/*kT* for the designed compounds.

To elaborate the luminescence dynamics of Mn^2+^ in MgGa_2_O_4_ lattices, the decay curves of the MgGa_2_O_4_:*x*Mn^2+^ phosphors were tested and depicted in Figure 3e. As demonstrated, all decay curves can be well fitted using a single exponential function:

(1) $$I\;\left(t\right)=A\;\exp\left(-t/\tau \right)$$

where *I(t)* is the luminescence intensities at given time *t*, *A* is a an amplitude of luminescence decay and *τ* corresponds to the excited state lifetime. As summarized in Figure S6a, the lifetime gradually shortens from 6.99 to 5.37 ms with increasing Mn^2+^ concentration from 0.0005 to 0.011, which can be attributed to enhanced nonradiative energy transfer among neighboring Mn^2+^ at high doping concentrations. This lifetime reduction further verifies the occurrence of concentration quenching in the Mn^2+^‐doped MgGa_2_O_4_ phosphors. Moreover, we also measured the decay curves of the Mg_0.999‐_ *_y_* Ga_2_O_4_:Mn^2+^ phosphors (Figure 3f), and they also can be fitted via a single exponential expression. Interestingly, the lifetime also gradually shortens with increasing Mg^2+^ deficiency (Figure S6b), indicating that the introduction of Mg^2+^ vacancies modifies the local electronic environment around Mn^2+^. The temperature‐dependent emission spectra of the MgGa_2_O_4_:0.001Mn^2+^ and Mg_0.939_Ga_2_O_4_:Mn^2+^ phosphors were measured over the temperature range of 303–503 K to evaluate their thermal stability (Figure 3g and Figure S7). The normalized emission spectra demonstrate that the emission peak spectral position remains almost unchanged with increasing temperature (Figure S8), which is highly desirable for temperature‐independent luminescent applications and optical manometry [15, 30]. Meanwhile, the resulting phosphors exhibit typical temperature‐induced luminescence quenching. Specifically, at 423 K, the fluorescence intensities of the MgGa_2_O_4_:0.001Mn^2+^ and Mg_0.939_Ga_2_O_4_:Mn^2+^ phosphors retain approximately 58.2% and 64.4% of their initial intensities at 303 K, respectively (see Figure 3h). Compared with the stoichiometric MgGa_2_O_4_:0.001Mn^2+^ phosphor, the Mg^2+^‐deficient phosphor exhibits an approximately 10.7% enhancement in thermal stability, confirming that nonstoichiometric engineering is an effective strategy for improving the thermal robustness of Mn^2+^‐doped MgGa_2_O_4_ phosphors. Additionally, the activation energy (Δ*E*) associated with thermal quenching was further calculated: [40, 41]

(2) $$I\left(T\right)=\frac{I_{0}}{1+A\exp\left(-\Delta E/kT\right)}$$

where the fluorescence intensities at 0 K and recorded temperature *T* are labeled by *I*_0_ and I(*T*), respectively, *A* refers to the fitting coefficient and *k* is the Boltzmann constant. Based on the linear fitting of ln(*I*_0_/*I*‐1) vs 1/*kT* (Figure 3i), the Δ*E* values the MgGa_2_O_4_:0.001Mn^2+^ and Mg_0.939_Ga_2_O_4_:Mn^2+^ phosphors are determined to be 0.30 and 0.29 eV, respectively. These results demonstrate that Mg^2+^ deficiency effectively improves the emission intensity and thermal stability of Mn^2+^‐doped MgGa_2_O_4_ phosphors, highlighting their great potential for multifunctional optical applications.

Beyond the photoluminescence properties, the synthesized phosphors also exhibit persistent luminescence behavior. To systematically evaluate their afterglow performance, the persistent luminescence decay curves of the MgGa_2_O_4_:0.001Mn^2+^ and Mg_0.939_Ga_2_O_4_:Mn^2+^ phosphors were recorded and presented in Figure 4a. As disclosed, both samples exhibit long‐lasting green afterglow emission after the removal of the excitation source. Specifically, the afterglow signal of the MgGa_2_O_4_:Mn^2+^ phosphor lasts around 400 s, while that of the Mg_0.939_Ga_2_O_4_:Mn^2+^ phosphor extends to nearly 800 s. Notably, Mg^2+^ deficiency regulation significantly enhances both the afterglow intensity and duration, indicating that nonstoichiometric engineering effectively improves the persistent luminescence performance. As is well documented, the afterglow features of the luminescent materials are highly related to the trap characteristics [42, 43]. Figure 4b presents the thermoluminescence (TL) curves of the MgGa_2_O_4_:0.001Mn^2+^ and Mg_0.939_Ga_2_O_4_:Mn^2+^ phosphors. Clearly, both samples exhibit very broad TL bands ranging from approximately 300 to 550 K, indicating the existence of multiple trap states within the MgGa_2_O_4_ lattice, as well as inherent defects, i.e., 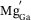 and ${\mathrm{Ga}}_{\mathrm{Mg}}^{o}$ anti‐site defects [37]. Compared with the stoichiometric MgGa_2_O_4_:0.001Mn^2+^ phosphor, the Mg_0.939_Ga_2_O_4_:Mn^2+^ phosphor presents significantly enhanced TL intensity, suggesting an increased concentration of trapping centers induced by Mg^2+^ deficiency. By analyzing these TL curves, one knows that they all can be fitted into multiple peaks (Figure S9), implying that the resulting phosphors exhibit continuous trap levels. In accordance with previous reports [37, 43], the trap depth (i.e., *E*) can be roughly evaluated via using the following function:

(3) $$E=T_{m}/500\;$$

where the peak temperature of the TL curve is labeled by *T*_m_. Consequently, the continuous trap depths were estimated, with the value ranging from 0.68 to 1.06 eV. In general, trap depth of 0.65‐0.80 eV is considered as shallow trap, whereas the value above 0.80 eV corresponds to deep trap. Thus, it can be concluded that the resulting phosphors possess a continuous trap distribution consisting of both shallow and deep traps, which is favorable for achieving efficient carrier storage and sustained electron release. Furthermore, the irradiation time dependent TL curves of the Mg_0.939_Ga_2_O_4_:Mn^2+^ phosphor were recorded so as to clarify the charging process, as depicted in Figure S10a. Significantly, the TL intensity gradually increases with prolonged irradiation time, indicating that the traps are progressively populated rather than instantaneously saturated. Additionally, we also systematically studied the release performance of electrons in the Mg_0.939_Ga_2_O_4_:Mn^2+^ phosphor. With increasing the heating rate (i.e., *β*), it is found that the maximum temperature of the TL glow curves (i.e., *T*_m_) gradually shifts to a high temperature region (see Figure S10b). Based on the variable heating rate method, the trap depth (*E*) and frequency factor (*s*) can be determined according to the following equation: [37, 44]

(4) $$\frac{\beta E}{kT_{m}^{2}}=s\;\exp\left(\frac{-E}{kT_{m}}\right)$$

where *s* refers to the frequency factor. The plot of ln(*T*_m_^2^/*β*) vs 1/*kT*_m_ (Figure 4c) exhibits a good linear relation, from which the trap depth and frequency factor are calculated to be 8.4 × 10^6^ s^−1^ and 0.56 eV, respectively. Moreover, the possibility (i.e., *P*) of electrons detrapping per unit time can be evaluated by the following function: [33, 44]

(5) $$P=s\;\exp\left(-E/kT\right)$$

**FIGURE 4:**
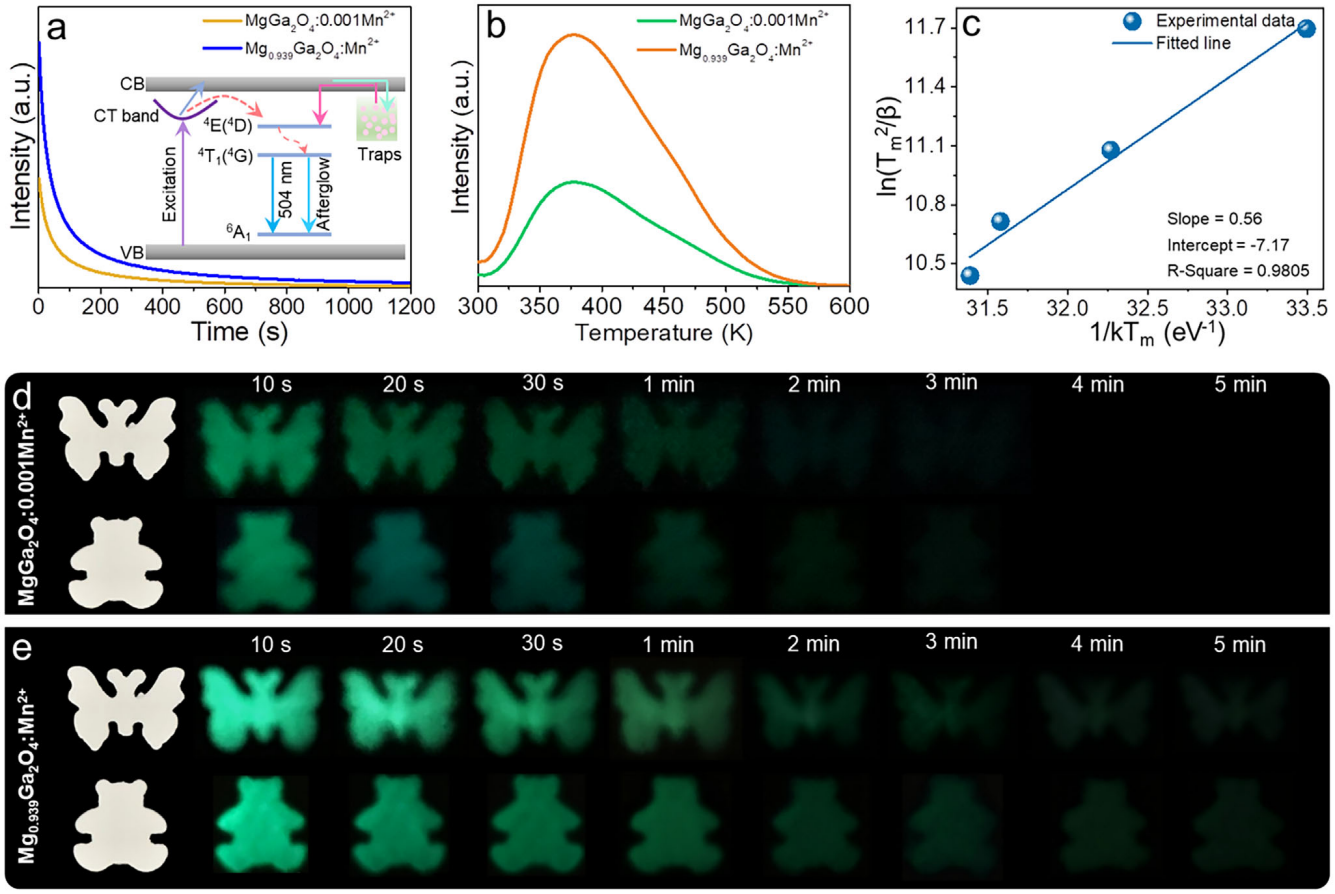
(a) Afterglow curves and (b) TL glow curves of the MgGa_2_O_4_:0.001Mn^2+^ and Mg_0.939_Ga_2_O_4_:Mn^2+^ phosphors. Inset of (a) shows the simplified energy level diagram for the designed compounds. (c) Plot of ln(T_m_^2^/*β*) vs 1/*k*T_m_ for the Mg_0.939_Ga_2_O_4_:Mn^2+^ phosphor. Optical images of information encryption prepared with (d) MgGa_2_O_4_:0.001Mn^2+^ and (e) Mg_0.939_Ga_2_O_4_:Mn^2+^ phosphors.

Consequently, the *p* value at room temperature is approximately 3.2 × 10^−3^ s^−1^. The relatively low escape probability indicates that the trapped electrons are stabilized at ambient conditions, thereby enabling prolonged afterglow emission. Based on the above results, a possible persistent luminescence mechanism is proposed, as illustrated in the inset of Figure 4a. A fraction of these excited electrons is captured by the defect‐related trap states generated in the lattice. After removing the excitation source, electrons stored in relatively shallow traps are gradually released and transferred back to the excited states of Mn^2+^, followed by radiative recombination and the generation of persistent green emission. The introduction of Mg^2+^ deficiency increases the density and optimizes the distribution of trapping centers, thereby improving the carrier storage capacity and afterglow performance. In the light of the luminescence features of the resultant phosphors, their applications in the optical anti‐counterfeiting are explored. By mixing the compounds (i.e., MgGa_2_O_4_:0.001Mn^2+^ and Mg_0.939_Ga_2_O_4_:Mn^2+^) with polydimethysiloxane (PDMS), we prepared different optical patterns, as depicted in Figure 4d,e. Irradiated by 254 nm UV light, these fabricated PDMS patterns emit intense green light. More importantly, after removing the excitation source, these PDMS patterns remain visible owing to the persistent luminescence behavior of the embedded phosphors, demonstrating their applicability for optical anti‐counterfeiting. Compared with the PDMS patterns fabricated using MgGa_2_O_4_:0.001Mn^2+^ phosphor, those containing Mg_0.939_Ga_2_O_4_:Mn^2+^ phosphor exhibit stronger emission intensity and longer‐lasting afterglow, further confirming the effectiveness of Mg^2+^ deficiency engineering in enhancing persistent luminescence performance. These results imply the deigned phosphors with good photoluminescence and afterglow features are promising candidates for optical anti‐counterfeiting.

### 3.2 High‐Pressure Studies

To ensure reliable application in luminescence‐based manometry, the developed phosphors should possess excellent structural stability under high‐pressure conditions. Herein, in‐situ high‐pressure XRD measurements were performed on the representative MgGa_2_O_4_:0.001Mn^2+^ and Mg_0.939_Ga_2_O_4_:Mn^2+^ phosphors to investigate their structural evolution under pressure, as shown in Figure 5a and Figure S11a, respectively. It should be noted that the diffraction peak positions observed in the high‐pressure XRD patterns are different from those presented in Figure 1, which originates from the different X‐ray wavelength employed in the measurements (i.e., Ag *Kα* radiation, *λ* = 0.4834 Å for high‐pressure XRD vs Cu Kα radiation, λ = 1.5406 Å for conventional XRD at ambient conditions). As displayed in Figure 5a, no disappearance or emergence of new diffraction peaks is observed upon compression, demonstrating that the Mg_0.939_Ga_2_O_4_:Mn^2+^ phosphor maintains a stable cubic phase within the investigated pressure range, i.e., up to 30.19 GPa. Similarly, the MgGa_2_O_4_:0.001Mn^2+^ phosphor also preserves its original crystal structure under high pressure (Figure S10a), demonstrating excellent high‐pressure structural robustness. Although no pressure‐induced phase transition occurs, the diffraction peaks gradually broaden and shift toward higher 2*θ* angles with increasing pressure. The peak broadening can be mainly attributed to enhanced lattice distortion and accumulated strain generated during compression, whereas the systematic peak shift toward higher angles originates from the pressure‐induced lattice contraction. Such effect may modify the local crystal‐field environment surrounding Mn^2+^, thereby providing the structural basis for pressure‐dependent spectral evolution, which will be discussed in the following part. Moreover, upon pressure release, all diffraction peaks gradually return to their original positions (Figure 5a and Figure S11a), suggesting excellent structural reversibility and recoverability of the investigated phosphors. Furthermore, to gain deeper insights into the pressure‐induced structural evolution, Rietveld refinements were further performed based on the high‐pressure XRD patterns of MgGa_2_O_4_:0.001Mn^2+^ and Mg_0.939_Ga_2_O_4_:Mn^2+^ phosphors (Figure 5b,c and Figure S11b,c). The refined diffraction profiles exhibit excellent agreement with the experimental data, confirming that the samples retain the cubic phase without detectable structural transformation throughout the compression process. Although the macroscopic phase structure remains unchanged under compression, significant variations in bond lengths, lattice parameters (i.e., *a* = *b* = *c*), and unit‐cell volumes are observed with increasing pressure, as illustrated in Figure 5d,e and Table S2, revealing continuous lattice compression induced by external pressure. Specifically, the unit cell volume of the MgGa_2_O_4_:0.001Mn^2+^ phosphor decreases from 637.0 to 607.5 Å^3^ within the pressure range of 0.49‐14.60 GPa (Table S4), while that of the Mg_0.939_Ga_2_O_4_:Mn^2+^ phosphor shrinks from 637.7 to 581.3 Å^3^ upon compression from ambient conditions up to 30.19 GPa (see Figure 5e and Table S4). These results demonstrate that the designed phosphors exhibit excellent structural stability and reversibility within the target pressure range, supporting their applicability under high‐pressure conditions.

**FIGURE 5:**
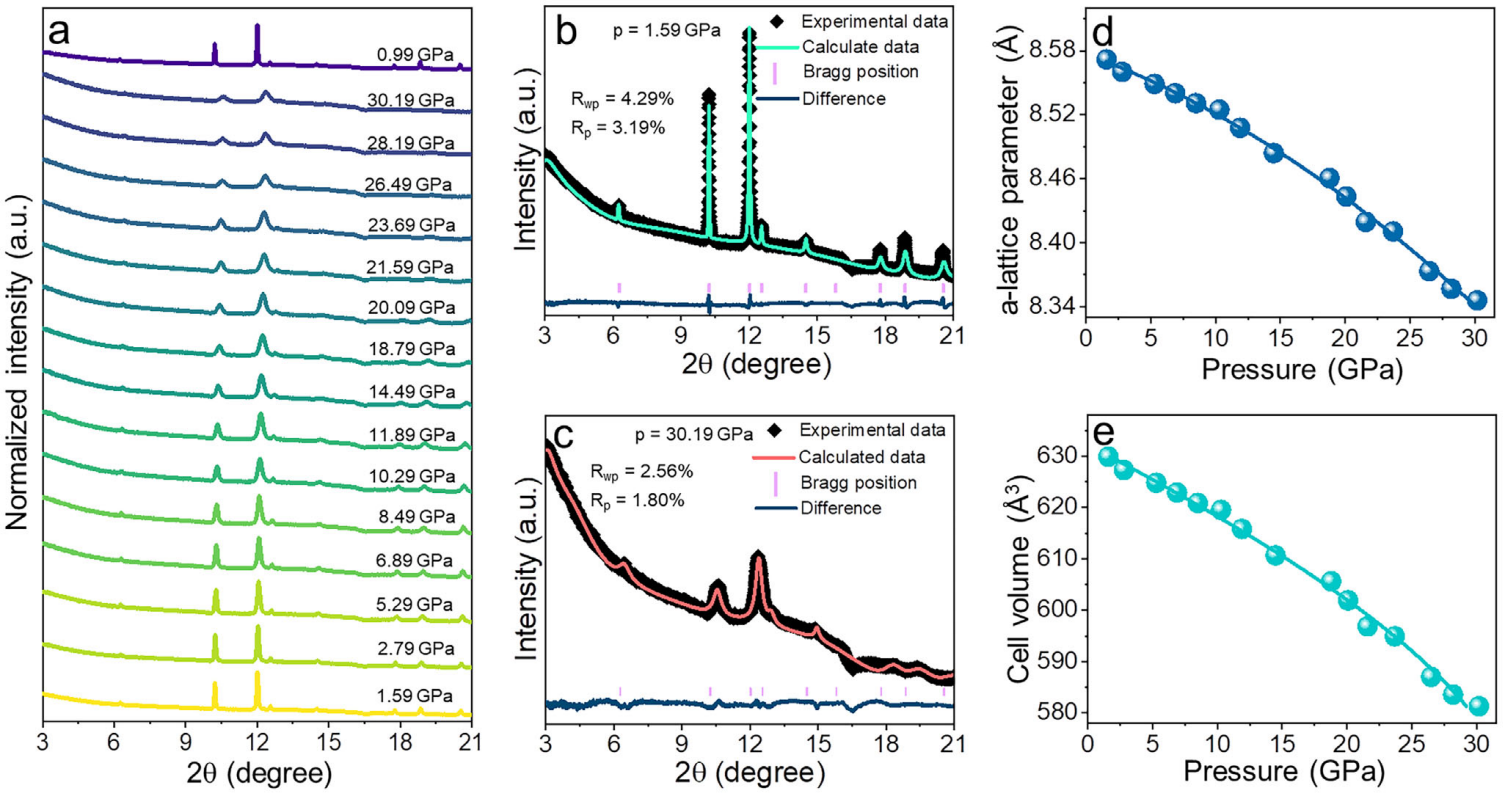
(a) Pressure‐dependent XRD profiles of the Mg_0.939_Ga_2_O_4_:Mn^2+^ phosphor. (b,c) Rietveld XRD refinements of the Mg_0.939_Ga_2_O_4_:Mn^2+^ phosphor recorded at (b) *p* = 1.59 and (c) *p* = 30.19 GPa. (d) Lattice parameters (i.e., *a = b = c*) and (e) unit cell volume of the Mg_0.939_Ga_2_O_4_:Mn^2+^ phosphor as a function of pressure.

As is well established, the emission of Mn^2+^ arising from d‐d electronic transition is highly sensitive to the surrounding crystal‐field environment, making Mn^2+^‐doped phosphors promising candidates for pressure‐responsive luminescence sensing [30, 31, 32, 33, 34, 35]. Herein, to investigate the pressure‐induced spectral evolution of the developed phosphors, their pressure‐dependent emission spectra were tested and the corresponding experimental setup is described in Figure 6a. Upon 266 nm UV light excitation, pressure‐dependent chromatic evolution is observed in the Mg_0.939_Ga_2_O_4_:Mn^2+^ phosphor. As the applied pressure increases from 0 to ≈30 GPa, the emitting color gradually changes from green to yellow (Figure 6b). Correspondingly, the CIE chromaticity coordinates continuously shifts from (0.098,0.643) to (0.561,0.437), which agrees well with the recorded optical images. Such a pronounced pressure‐induced change of the emission color makes the Mg_0.939_Ga_2_O_4_:Mn^2+^ phosphor a promising candidate for visual optical manometry. Figure 6c shows the normalized emission spectra of the Mg_0.939_Ga_2_O_4_:Mn^2+^ phosphor as a function of pressure. Despite undergoing severe lattice compression, the characteristic Mn^2+^ emission remains clearly detectable throughout the entire pressure range. Moreover, with an increase in pressure, the resultant phosphors present an evident spectral red‐shift (Figure 6c,d), where its emission band centroid shifts from 509.2 to 602.9 nm (displacement = 93.7 nm). Note that, the emission centroid fully recovers to its original position after pressure release (Figure S12), indicating excellent structural reversibility and luminescence recovery. On the basis of the previous reports [30, 31, 32, 33, 34, 35], the pressure‐induced red‐shift of the Mn^2+^ emission can be mainly attributed to the combined effects of an enhanced nephelauxetic effect and increased crystal‐field strength (leading to improved splitting of the multiplets) in the compressed structure. Upon compression, the lattice shrinkage takes place, which reduces the bond length, and thus, the energy of the Mn^2+^ emitting level decreases, resulting in the observed spectral red‐shift with pressure. Furthermore, when high pressure is applied, the interionic distance (i.e., Mn^2+^–O^2−^) is shortened and the bond covalency is improved, leading to a reduction in the free‐ion parameters (i.e., Coulombic and spin orbit) due to the enhanced nephelauxetic effect [30, 31, 32, 33, 34, 35]. Consequently, compression of the material results in a decrease in energy difference between the ground and excited states, leading the observed red‐shift of the emission band under pressure. The synergistic contribution of the aforementioned factors is therefore responsible for the remarkable spectral tuneability under extreme conditions.

**FIGURE 6:**
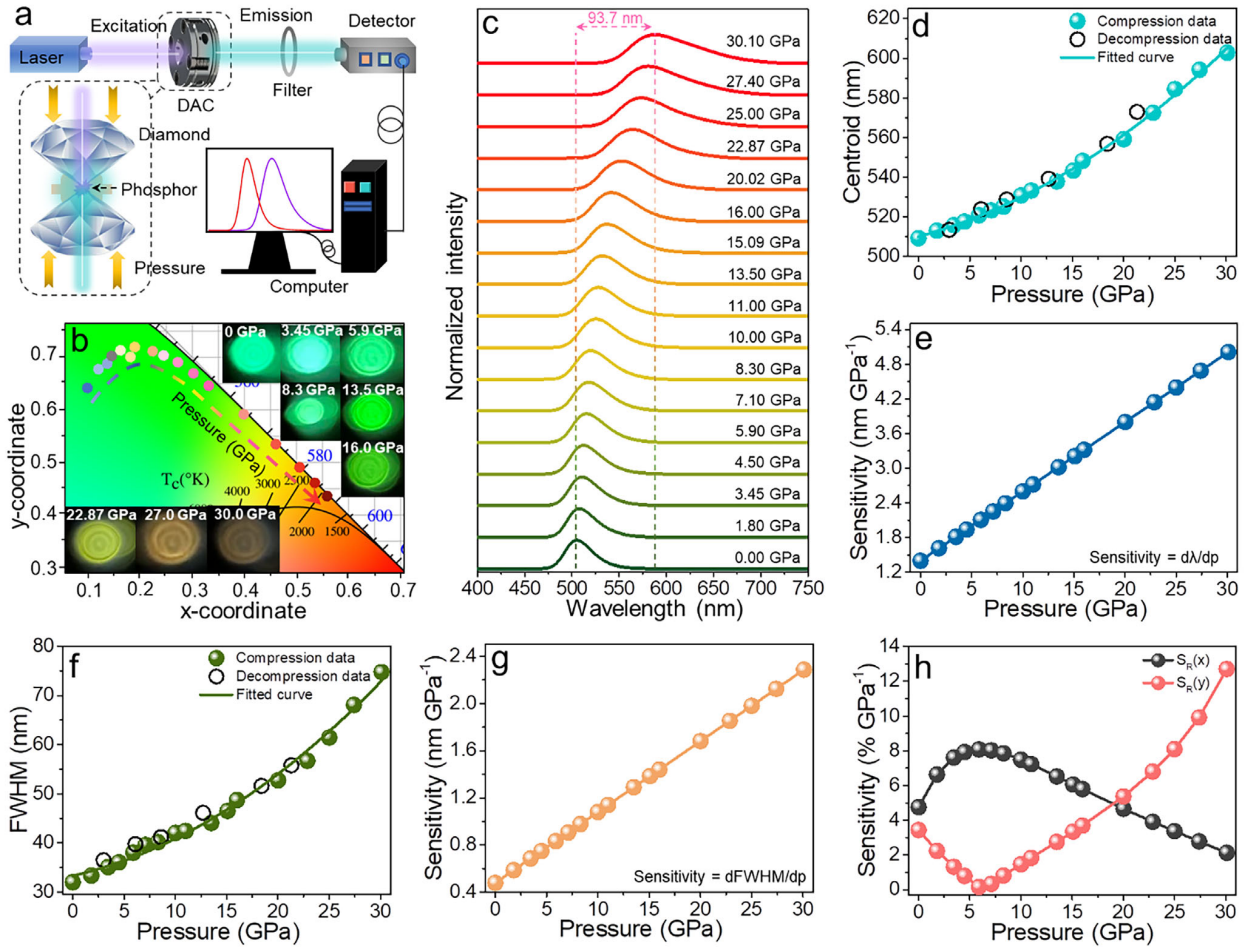
(a) Schematic diagram of the experimental setup used for the high‐pressure luminescence measurements. (b) CIE chromaticity diagram showing the pressure evolution of the color coordinates for the Mg_0.939_Ga_2_O_4_:Mn^2+^ material, together with the photographs of its luminescence in a DAC with increasing pressure. (c) Pressure‐dependent emission spectra; (d) determined emission band centroid and (e) the corresponding sensitivity (d*λ*/d*p*) as a function of pressure; (f) determined FWHM value and (g) the corresponding sensitivity (dFWHM/d*p*), as well as (h) the color coordinates‐based relative sensitivity (*S*_R_) values for the Mg_0.939_Ga_2_O_4_:Mn^2+^ material as a function of pressure.

Figure 6d presents pressure evolution of the emission band centroid of the Mg_0.939_Ga_2_O_4_:Mn^2+^ phosphor. As disclosed, the relation between pressure and emission band centroid (i.e., *λ*) can be described well via a simple second‐order polynomial expression (empirical model), i.e., λ  =  0.06*p* ^2^ + 1.40*p* + 510.05. Thereby, the wavelength‐shift sensitivity (i.e., d*λ*/d*p*) was calculated and presented in Figure 6e. With increasing pressure, the sensitivity gradually increases and reaches a maximum value of approximately 5.01 nm GPa^−1^ at 30.10 GPa. Interestingly, the obtained sensitivity is not only much higher than these conventional luminescent pressure sensors, i.e., ruby (d*λ*/d*p* = 0.365 nm GPa^−1^) and SrB_4_O_7_:Sm^2+^ (d*λ*/d*p* = 0.25 nm GPa^−1^), but also comparable with the majority of recent reports on the development of various highly sensitive optical manometers (see Table 1), which are promising alternatives for the commonly used ruby sensors. Furthermore, it can be seen that the operational pressure range of the Mg_0.939_Ga_2_O_4_:Mn^2+^ phosphor extends over 30 GPa, exceeding that of many recently reported luminescent manometers, as displayed in Table 1. These results indicate that the developed optical pressure sensors, endowed with high sensitivity and broad operating pressure range, are promising candidates for modern luminescent manometry via utilizing the emission band centroid as pressure indicator.

**TABLE 1:** Comparison of the pressure sensing properties of the resulting phosphors with previously developed luminescent manometers.

Compounds	Sensitivity (nm GPa^−1^)		Operating range (GPa)	λ_em_ (nm)	Refs.
	d*λ*/d*p*	dFWHM/d*p*			
ZnAl_2_O_4_:Mn^2+^	2.12	0.71	0–30.70	519.1	[15]
BaLi_2_Al_2_Si_2_N_6_:Eu^2+^	1.58	—	0.101–20.00	532	[25]
Lu_3_Al_5_O_12_:Eu^3+^/Mn^4+^	0.86	—	0.00–19.82	666.61	[26]
KGa_5_S_8_:Eu^2+^	14.49	4.2	≈0.00–10.44	541.4	[27]
Ca_8_Zn(SiO_4_)_4_Cl_2_:Eu^2+^	4.18	—	0.46–20.35	500.2	[28]
MgAl_2_O_4_:Mn^2+^	2.36	1.29	0.24–14.65	526.5	[30]
Zn_2_GeO_4_:Mn^2+^	21.3	17.0	0.00–6.76	535.9	[31]
NaY_9_(SiO_4_)_6_O_2_:Mn^2+^	7.00	10.13	0.75–7.16	617.2	[32]
Lu_2_Mg_2_Al_2_Si_2_O_12_:Mn^2+^	3.53	—	0.00–25.60	597.61	[33]
Mg_2_Y_8_(SiO_4_)_6_O_2_:Mn^2+^	4.75	—	0.13–10.89	613.8	[34]
Ca_2_Gd_8_(SiO_4_)_6_O_2_:Mn^2+^	7.25	—	0.38–13.66	592.2	[35]
Cs_2_ZrCl_6_:Te^4+^	6.30	1.86	0.13–20.61	582.1	[40]
NaMgBO_3_:Ce^3+^	2.94	—	0.31–17.18	469.3	[45]
MgGa_2_O_4_:0.001Mn^2+^	2.46	0.65	0.11–13.41	509.8	This work
Mg_0.939_Ga_2_O_4_:Mn^2+^	5.01	2.29	0.00–30.10	509.2	This work

Apart from the emission band shift, the pressure‐triggered emission band broadening is also observed. Apparently, the full width at half maximum (FWHM) of the Mn^2+^ emission band continuously increases from 30.0 to 74.8 nm, as pressure increases from 0 to 30.10 GPa (Figure 6f). Moreover, after decompression, the FWHM value returns to its initial state, confirming the reversible pressure response of the material studied. By investigating the relationship between the FWHM and pressure, it was found that they follow a second‐order polynomial dependence, i.e., FWHM  =  0.03*p* ^2^ + 0.458*p* + 33.33 (see Figure 6f). Accordingly, the corresponding FWHM‐based sensitivity, i.e., dFWHM/d*p*, was determined and illustrated in Figure 6g. With pressure elevation, a gradual increase in the dFWHM/d*p* value is observed, reaching a maximum of 2.29 nm GPa^−1^ at 30.10 GPa. Compared with previously reported luminescent pressure sensors (Table 1), this value is highly competitive, suggesting that the Mg_0.939_Ga_2_O_4_:Mn^2+^ phosphor can also serve as an effective FWHM‐based optical manometer. In addition, the compression‐decompression cycling experiment (Figure S13) demonstrates excellent signal reproducibility and structural reversibility, further confirming the reliability of the developed phosphor for optical manometry.

As demonstrated in Figure 6b, the emission color of the studied samples undergoes remarkable evolution upon compression, endowing its potential for colorimetric pressure sensing. To quantitatively evaluate the pressure‐induced chromatic variation, the chromaticity shift (Δ*s*) was calculated according to the following equation: [45, 46]

(7) $$\Delta s=\sqrt{{\left(u_{m}^{\prime }-u_{0}^{\prime }\right)}^{2}+{\left(v_{m}^{\prime }-v_{0}^{\prime }\right)}^{2}+{\left(w_{m}^{\prime }-w_{0}^{\prime }\right)}^{2}}\;$$

where *u*′ = 4*x*/(3 − 2*x* + 12*y*) , *v*′ = 9*y*/(3 − 2*x* + 12*y*)  and *w*′ =  1 − *u*′ − *v*′, of which the subscripts *m* and 0 represent the color coordinates at the measured pressure and initial pressure, respectively. The pressure‐dependent Δ*s* values of the Mg_0.939_Ga_2_O_4_:Mn^2+^ phosphor is shown in Figure S14. Evidently, a pronounced chromatic shift is observed throughout the compression process, confirming the high sensitivity of the emission color toward external pressure. This remarkable color evolution enables the developed phosphors to serve as colorimetric pressure sensors. By analyzing the pressure‐dependent color coordinates, i.e., the *x*‐ and *y*‐coordinates, it was found that both follow a third‐order polynomial relationship, namely, *x*  =   − 1.512 × 10^−5^ *p* ^3^ + 8.009 × 10^−4^ *p* ^2^ + 0.005*p* + 0.105 and *y*  =  2.641 × 10^−5^ *p* ^3^ − 0.002*p* ^2^ + 0.022*p* + 0.639 (Figure S15). Furthermore, to further quantify the colorimetric sensing capability, the relative sensitivity (i.e., *S_R_*) was calculated, as defined below: [34, 35]

(8) $$S_{R}=\left|\frac{1}{\mathrm{CIE}}\frac{d\mathrm{CIE}}{dp}\right|\;\times 100\%$$

where CIE refers to the color coordinate and the calculated relative sensitivities based on *x*‐ and *y*‐coordinates are denoted as *S_R_* (*x*) and *S_R_* (*y*), respectively. The determined *S_R_* (*x*) and *S_R_* (*y*) values of the Mg_0.939_Ga_2_O_4_:Mn^2+^ phosphor are depicted in Figure 6h, with maximum values of 8.08% GPa^−1^ and 12.71% GPa^−1^, respectively. Compared with previously reported colorimetric luminescent pressure sensors, such as ZnAl_2_O_4_:Mn^2+^ (*S_R_* (*x*) = 6.14% GPa^−1^ and *S_R_* (*y*) = 18.34% GPa^−1^), Ca_8_Zn(SiO_4_)_4_Cl_2_:Eu^2+^ (*S_R_* (*x*) = 9.53% GPa^−1^ and *S_R_* (*y*) = 13.78% GPa^−1^), Mg_2_Y_8_(SiO_4_)_6_O_2_:Mn^2+^ (*S_R_* (*x*) = 1.59% GPa^−1^ and *S_R_* (*y*) = 3.27% GPa^−1^), Ca_2_Gd_8_(SiO_4_)_6_O_2_:Mn^2+^ (*S_R_* (*x*) = 12.64% GPa^−1^ and *S_R_* (*y*) = 3.25% GPa^−1^), NaMgBO_3_:Ce^3+^ (*S_R_* (*x*) = 1.87% GPa^−1^ and *S_R_* (*y*) = 6.22% GPa^−1^), etc. [15, 28, 34, 35, 45], the determined *S_R_* values are satisfactory, demonstrating the feasibility of the resultant phosphors for colorimetric pressure sensing. Therefore, by employing multiple luminescence parameters, including emission band centroid, FWHM and chromaticity coordinates as pressure indicators, the Mn^2+^‐doped MgGa_2_O_4_ phosphors can be used for high performance, multi‐modal luminescence manometry.

As clarified above, the photoluminescence and afterglow properties of the stoichiometric phosphors can be effectively improved via nonstoichiometric engineering (i.e., by Mg^2+^ deficiency). However, whether the Mg^2+^ deficiency strategy can further optimize the pressure‐sensing capability remains unclear. To address this issue, the pressure‐sensing capacities of the stoichiometric MgGa_2_O_4_:0.001Mn^2^^+^ phosphor were systematically investigated, as well. From the pressure‐dependent emission spectra (see Figure S16a), the expected spectral red‐shift of the emission band for MgGa_2_O_4_:0.001Mn^2+^ phosphor was observed. The observed shift was reversible, as confirmed by the decompression data presented in Figure S16b. Based on the pressure‐dependent emission spectra, the corresponding color coordinates were determined and presented in the CIE chromaticity diagram (Figure S16c). The emission band centroid extracted from the emission spectra shifts from 509.8 to 534.9 nm within the pressure range of 0.11–13.41 GPa (Figure S16d). Moreover, the relation between emission band centroid and pressure obeys a second‐order polynomial formula, that is, λ  =   − 0.04*p* ^2^ + 2.47*p* + 508.73. Accordingly, the wavelength‐shift sensitivity (d*λ*/d*p*) was determined, reaching a maximum value of 2.46 nm GPa^−1^ at the lowest pressure value (Figure S16e). Meanwhile, pressure‐induced broadening of the Mn^2+^ emission band is also observed. The FWHM value increases from 32.9 to 39.7 nm upon compression from 0.11 to 13.41 GPa (Figure S16f), of which their relation can be described as: FWHM  =   − 0.01*p* ^2^ + 0.649*p* + 32.95. By using FWHM as manometric parameter, the sensitivity (i.e., dFWHM/d*p*) of the studied sample was determined, and its maximum value is 0.65 nm GPa^−1^ (see Figure S16g). It is worth noting here, that in contrast to the Mg^2+^‐deficient material (where the both sensitivities increased with pressure), in this case both sensitivities (shift‐ and FWHM‐based) decreased with pressure, revealing the opposite tendencies, which indicates clear advantages for the optical sensing with non‐stoichiometric material.

In addition, the pressure‐dependent color coordinates of the MgGa_2_O_4_:0.001Mn^2+^ phosphor can also be fitted via a third‐order polynomial function, namely, for *x*‐coordinate, it can be expressed as *x*  =   − 6.848 × 10^−4^ *p* ^3^ + 0.002*p* ^2^ − 6.848 × 10^−4^ *p* + 0.06 (Figure S16h), while it changes to *y*  =  1.024 × 10^−4^ *p* ^3^ − 0.004*p* ^2^ + 0.045*p* + 0.583, for *y*‐coordinate (Figure S16i). Consequently, the maximum *S_R_* (*x*) and *S_R_* (*y*) values for the MgGa_2_O_4_:Mn^2+^ phosphor are 14.07% and 7.67% GPa^−1^, respectively, as shown in Figure S16j. Note that, the cyclic compression‐decompression measurements confirm the excellent reversibility and stability of the MgGa_2_O_4_:Mn^2+^ phosphor (see Figure S16k).

A comparison between MgGa_2_O_4_:0.001Mn^2+^ and Mg_0.939_Ga_2_O_4_:Mn^2+^ phosphors clearly demonstrates the superiority of Mg^2+^ deficiency engineering in optimizing the optical pressure sensing performance. Specifically, when emission band centroid and FWHM are adopted as the manometric parameters, the Mg_0.939_Ga_2_O_4_:Mn^2+^ phosphor exhibits significantly enhanced sensitivities compared with the MgGa_2_O_4_:0.001Mn^2+^ phosphor, i.e., the maximal d*λ*/d*p* and dFWHM/d*p* values for the Mg^2+^‐deficient phosphor are approximately 2.04 and 3.52 times higher than those for the stoichiometric counterpart, respectively. These results unambiguously demonstrate that non‐stoichiometric engineering effectively amplifies pressure sensitivity of the Mg^2+^‐deficient MgGa_2_O_4_:Mn^2+^ sensor material and improves its overall luminescence performance, making it a promising candidate for optical manometry.

The enhanced luminescence performance induced by Mg^2+^ deficiency can be caused by defect‐mediated carrier trapping and modifications of the local coordination environment of Mn^2+^ ions. In the cubic spinel structure, Mg^2+^ deficiency deviates the local environment and is accompanied by a lattice contraction, as evidenced by the Rietveld refinements (Table S1). These structural changes can modify the crystal field around the Mn^2+^ activators and their interactions with the lattice, thereby influencing the radiative and nonradiative relaxation mechanisms. The material with Mg^2+^ deficit, i.e., Mg_0.939_Ga_2_O_4_:Mn^2+^ phosphor exhibits approximately 1.6 times more intense emission than the stoichiometric material, while the emission band centroid and FWHM remain nearly unchanged, suggesting that the major effect is an increase in emission efficiency rather than a substantial change in the distribution of emitting Mn^2+^ environments. Mg^2+^ deficiency also enhances the thermoluminescence intensity and extends the afterglow duration from approximately 400 to 800 s, indicating an increased population of defect‐related carrier traps and more effective carrier storage and release. Although the precise microscopic identities and charge‐compensation mechanisms of these defects cannot be established from the present measurements alone, the results support a defect‐mediated mechanism in which nonstoichiometry simultaneously modifies the local environment of Mn^2+^ and the trap‐state distribution. Under external pressure, the resulting lattice compression further shortens the Mn^2+^–O^2−^ distance and enhances the crystal‐field strength and metal–ligand covalency, producing a pronounced redshift and broadening of the emission band. The enhanced pressure sensitivity of the Mg‐deficient phosphor therefore suggests that nonstoichiometric defect engineering modifies the pressure‐dependent optical response of the Mn^2+^ ions, providing a common structural basis for the improved photoluminescence, persistent luminescence, and optical manometry performance.

## 4 Conclusions

In summary, Mn^2+^‐doped MgGa_2_O_4_ phosphors were prepared through a high‐temperature solid‐state reaction method and their luminescence performances were systematically regulated through nonstoichiometric engineering and external pressure modulation. Excited by UV light the resulting phosphors can emit intense green emission from Mn^2+^ d‐d transition, of which the optimal doping content of Mn^2+^ in MgGa_2_O_4_ lattice is 0.1 mol%. Benefiting from Mg^2+^ deficiency engineering, the photoluminescence intensity, thermal stability and persistent luminescence performance of the studied samples are simultaneously enhanced. The improved afterglow characteristics induced by nonstoichiometric route, enabled their applications in optical anti‐counterfeiting.

Furthermore, the in‐situ high‐pressure XRD patterns indicate that the synthesized phosphors present excellent structural stability within the investigated pressure range. The pressure‐induced lattice contraction effectively modifies the local crystal‐field environment surrounding Mn^2+^, resulting in remarkable pressure‐dependent spectral evolution. Notably, compared with the stoichiometric compounds, the Mg^2+^ deficient material exhibits significantly enhanced pressure sensitivity and improved overall sensing performance. Particularly, through analyzing the responses of the emission band centroid and FWHM to pressure, the pressure sensitivities of the Mg_0.939_Ga_2_O_4_:Mn^2+^ phosphor are 5.01 and 2.29 nm GPa^−1^, respectively, with a broad working pressure range up to 30.10 GPa. Additionally, colorimetric optical manometry is realized through pressure‐dependent chromaticity evolution, providing a maximum *S_R_* value of 12.71% GPa^−1^, and allowing visual pressure monitoring. Our findings may provide new insights into defect‐mediated regulation of crystal‐field‐sensitive luminescent materials and offers a viable pathway toward multifunctional optical sensing systems.

## Author Contributions

**Peng Du**: formal analysis, supervision, data curation, software, methodology, Writing – original draft, Writing – review and editing, conceptualization, visualization. **Yifei Yu**: investigation, visualization, formal analysis, data curation. **Yue Guo**: data curation, software, investigation, methodology, validation. **Przemysław Woźny**: visualization, methodology, validation, writing – review and editing. **Shualing Ma**: funding acquisition, writing – review and editing, project administration, supervision, conceptualization. **Laihui Luo**: supervision, writing – review and editing, validation, funding acquisition, resources. **Marcin Runowski**: writing – review and editing, project administration, resources, supervision.

## Conflicts of Interest

The authors declare no conflicts of interest.

## Supporting information




**Supporting File**: advs78629‐sup‐0001‐SuppMat.docx.

## Data Availability

The data that support the findings of this study are available from the corresponding author upon reasonable request.
