# Supplementary material for: Nonstoichiometric Defect Engineering in Mn2+‐Doped MgGa2O4 Persistent Phosphors Toward Enhanced Luminescence and Multimodal Optical Manometry
Source: Adv Sci (Weinh). 2026 Nov 3:e78629. Online ahead of print. doi: 10.1002/advs.78629 (PMC13731076; doi:10.1002/advs.78629)
Supplement: Supplementary file 1 [file ADVS-9999-e78629-s001.docx]

**Supporting Information for:**

**Nonstoichiometric defect engineering in Mn^2+^-doped MgGa_2_O_4_ persistent phosphors toward enhanced luminescence and multimodal optical manometry**

Peng Du^a,*^, Yifei Yu^a^, Yue Guo^b^, Przemysław Woźny^c^, Shualing Ma^a^, Laihui Luo^a,*^, Marcin Runowski^c,*^

*^a^School of Physical Science and Technology, Ningbo University, 315211 Ningbo, Zhejiang, China*

*^b^School of Applied Physics and Materials, Wuyi University, Jiangmen, Guangdong, 529020, China*

*^c^Adam Mickiewicz University, Faculty of Chemistry, Uniwersytetu Poznańskiego 8, 61-614 Poznań, Poland*

**Corresponding authors:**

**E-mail:** dupeng@nbu.edu.cn or dp2007good@sina.com (P. Du); luolaihui@nbu.edu.cn (L. Luo); runowski@amu.edu.pl (M. Runowski)

**Table S1** Lattice parameters of the Mg_0.999-_*_y_*Ga_2_O_4_:Mn^2+^ (*i.e*., *y* = 0.00, 0.02, 0.04, 0.06, 0.08 and 0.10) phosphors.

| **Parameter** | ***y* value** | | | | | |
| --- | --- | --- | --- | --- | --- | --- |
|  | **0.00** | **0.02** | **0.04** | **0.06** | **0.08** | **0.10** |
| *a* = *b* = *c* (Å) | 8.26207 | 8.26138 | 8.26084 | 8.26016 | 8.25961 | 8.25889 |
| Volume (Å^3^) | 563.983 | 563.843 | 563.732 | 563.593 | 563.480 | 563.333 |
| *R_p_* | 13.69% | 18.932% | 16.674% | 14.10% | 16.682% | 17.982% |
| *R_wp_* | 17.38% | 13.74% | 12.10% | 18.32% | 11.97% | 13.00% |
| χ^2^ | 1.89 | 1.33 | 1.41 | 1.95 | 1.33 | 1.32 |

**Table S2** The elemental analysis of the MgGa_2_O_4_:0.001Mn^2+^ and Mg_0.939_Ga_2_O_4_:Mn^2+^ phosphors.

| **MgGa_2_O_4_:0.001Mn^2+^** | | | **Mg_0.939_Ga_2_O_4_:Mn^2+^** | | |
| --- | --- | --- | --- | --- | --- |
| **Elements** | **Atomic percentage** | | **Elements** | **Atomic percentage** | |
|  | **Theory (%)** | **EDS (%)** |  | **Theory (%)** | **EDS (%)** |
| Mg | 14.27% | 18.89% | Mg | 13.53% | 17.70% |
| Ga | 28.57% | 34.89% | Ga | 28.82% | 36.05% |

The elemental compositions of the MgGa_2_O_4_:0.001Mn^2+^ and Mg_0.939_Ga_2_O_4_:Mn^2+^ phosphors were characterization by EDS with ZAF correction. Accordingly, for MgGa_2_O_4_:0.001Mn^2+^ phosphor, the Mg/Ga ratio is determined to be around 0.5414, while it decreases to 0.4909 in Mg_0.939_Ga_2_O_4_:Mn^2+^ phosphor. The measured Mg/Ga ratios decrease gradually with the increase of designed Mg deficiency, following the trend of precursor modulation.

**Table S3** FWHM of the emission band in MgGa_2_O_4_:*x*Mn^2+^ and Mg_0.999-_*_y_*Ga_2_O_4_:Mn^2+^ phosphors

| ***x* value** | **FWHM** | ***y* value** | **FWHM** |
| --- | --- | --- | --- |
| 0.0005 | 30.0 nm | 0.02 | 30.1 nm |
| 0.001 | 30.2 nm | 0.04 | 29.9 nm |
| 0.003 | 29.9 nm | 0.06 | 29.8 nm |
| 0.005 | 29.7 nm | 0.08 | 29.8 nm |
| 0.007 | 29.8 nm | 0.10 | 29.3 nm |
| 0.009 | 30.2 nm |  |  |
| 0.011 | 30.3 nm |  |  |

**Table S4** Lattice parameters and cell volume of the MgGa_2_O_4_:0.001Mn^2+^ and Mg_0.939_Ga_2_O_4_:Mn^2+^ phosphors as a function of pressure.

| **MgGa_2_O_4_:0.001Mn^2+^** | | | **Mg_0.939_Ga_2_O_4_:Mn^2+^** | | |
| --- | --- | --- | --- | --- | --- |
| **Pressure** | ***a = b = c*** | **volume** | **Pressure** | ***a = b = c*** | **Volume** |
| 0.49 GPa | 8.60427 Å | 637.004 Å^3^ | 1.59 GPa | 8.572046 Å | 629.874 Å^3^ |
| 1.69 GPa | 8.58297 Å | 632.285 Å^3^ | 2.79 GPa | 8.560293 Å | 627.286 Å^3^ |
| 2.79 GPa | 8.51977 Å | 618.42 Å^3^ | 5.29 GPa | 8.548994 Å | 624.806 Å^3^ |
| 4.79 GPa | 8.51544 Å | 617.477 Å^3^ | 6.89 GPa | 8.54027 Å | 622.896 Å^3^ |
| 5.49 GPa | 8.51142 Å | 616.604 Å^3^ | 8.49 GPa | 8.53097 Å | 620.862 Å^3^ |
| 8.29 GPa | 8.5115 Å | 616.62 Å^3^ | 10.29 GPa | 8.52475 Å | 619.504 Å^3^ |
| 9.30 GPa | 8.50675 Å | 615.589 Å^3^ | 11.89 GPa | 8.507783 Å | 615.814 Å^3^ |
| 11.00 GPa | 8.50299 Å | 614.774 Å^3^ | 14.49 GPa | 8.48396 Å | 610.655 Å^3^ |
| 12.00 GPa | 8.49308 Å | 612.625 Å^3^ | 18.79 GPa | 8.460583 Å | 605.621 Å^3^ |
| 13.80 GPa | 8.48266 Å | 610.373 Å^3^ | 20.09 GPa | 8.443289 Å | 601.915 Å^3^ |
| 14.60 GPa | 8.47934 Å | 609.659 Å^3^ | 21.59 GPa | 8.41952 Å | 596.846 Å^3^ |
|  |  |  | 23.69 GPa | 8.410533 Å | 594.936 Å^3^ |
|  |  |  | 26.49 GPa | 8.37314 Å | 587.036 Å^3^ |
|  |  |  | 28.19 GPa | 8.356644 Å | 583.574 Å^3^ |
|  |  |  | 30.19 GPa | 8.345832 Å | 581.31 Å^3^ |

**Figure S1** Rietveld XRD refinement results of the (a) Mg_0.979_Ga_2_O_4_:Mn^2+^, (b) Mg_0.959_Ga_2_O_4_:Mn^2+^, (c) Mg_0.919_Ga_2_O_4_:Mn^2+^ and (d) Mg_0.899_Ga_2_O_4_:Mn^2+^ phosphors.

**Figure S2** (a) Full-survey XPS spectrum of the MgGa_2_O_4_:Mn^2+^ phosphor. High-resolution XPS spectra of (b) Mg 2p, (c) Ga 2p and (d) O 1s in MgGa_2_O_4_:0.001Mn^2+^ phosphor

**Figure S3** SEM images of the MgGa_2_O_4_:*x*Mn^2+^ phosphors doped with the Mn^2+^ contents of (a) *x* = 0.0005, (b) *x* = 0.003, (c) *x* = 0.005, (d) *x* = 0.007, (e) *x* = 0.009 and (f) *x* = 0.011.

**Figure S4** (a) TEM image, (b) high-resolution TEM image, (c) SAED pattern, (d) EDS spectrum and (e)-(h) elemental mapping results of the MgGa_2_O_4_:0.001Mn^2+^ phosphor.

**Figure S5** Plot of log(I/*x*) vs. log(*x*) for the MgGa_2_O_4_:*x*Mn^2+^ phosphors.

To study the involved concentration quenching mechanism, the critical distance (*R_c_*) of Mn^2+^ in MgGa_2_O_4_ lattice is calculated, as defined below: ^[1]^

$R_{c}=2\left( \frac{3V}{4Z\pi x_{c}} \right)^{1/3}$	(1)

where *V* stands for the cell volume, *x_c_* denotes the doping concentration and *Z* is the number of cation in a unit cell. Here, the *V*, *x_c_* and *Z* values are 563.98 Å^3^, 0.001 and 8, respectively. As a consequence, the *R_c_* value is determined to be 51.3 Å, which is larger than 5 Å, indicating that the multipolar interaction can be responsible for concentration quenching mechanism. Moreover, on the basis of the relation between fluorescence intensity (I) and doping concentration (*x*), we further analyzed the concentration quenching mechanism in the studied samples, as follows:^[2]^

$\log\left( \frac{I}{x} \right)=A-\frac{\theta}{3}\log\left( x \right)$	(2)

where *A* is constant and the θ = 6, 8 and 10 is assigned to the electric dipole-dipole, dipole-quadrupole and quadrupole-quadrupole interactions, respectively. Accordingly to the experimental data, it can be seen that the slope of the fitted line is -1.49, leading to the θ value of 4.47, which is close to 6. Therefore, it is reasonable to consider that the concentration quenching mechanism in the MgGa_2_O_4_:*x*Mn^2+^ phosphors results from electric dipole-dipole interaction.

**Figure S6** Lifetime of (a) MgGa_2_O_4_:*x*Mn^2+^ and Mg_0.999_*_-y_*Ga_2_O_4_:Mn^2+^ phosphors at ambient conditions.

**Figure S7** Temperature-dependent emission spectra of the MgGa_2_O_4_:Mn^2+^ phosphor.

**Figure S8** Normalized temperature-dependent emission spectra of (a) MgGa_2_O_4_:Mn^2+^ and (b) Mg_0.939_Ga_2_O_4_:Mn^2+^ phosphors.

**Figure S9** TL glow curves of (a) MgGa_2_O_4_:Mn^2+^ and (b) Mg_0.939_Ga_2_O_4_:Mn^2+^ phosphors.

**Figure S10** TL glow curves of the Mg_0.939_Ga_2_O_4_:Mn^2+^ phosphor recorded at different (a) irradiation time and (b) heating rate.

**Figure S11** (a) Pressure-dependent XRD profiles of the MgGa_2_O_4_:Mn^2+^ phosphor. Rietveld XRD refinements of the MgGa_2_O_4_:Mn^2+^ phosphor recorded at (b) *p* = 0.49 and (c) *p* = 14.60 GPa.

**Figure S12** Emission spectra of the Mg_0.939_Ga_2_O_4_:Mn^2+^ phosphor during the decompression process.

**Figure S13** Emission spectra of Mg_0.939_Ga_2_O_4_:Mn^2+^ phosphor as a function of pressure.

**Figure S14** Pressure-dependent Δ*s* value of the Mg_0.939_Ga_2_O_4_:Mn^2+^ phosphor.

**Figure S15** Pressure-dependent (a) *x*-coordinate and (b) *y*-coordinate of the Mg_0.939_Ga_2_O_4_:Mn^2+^ phosphor.

**Figure S16** Emission spectra of the MgGa_2_O_4_:Mn^2+^ phosphor during (a) compression and (b) decompression. (c) CIE chromaticity diagram of the MgGa_2_O_4_:Mn^2+^ phosphor as a function of pressure. (d) Emission band centroid, (e) sensitivity (*i.e*., d*λ*/d*p*), (f) FWHM value, (g) sensitivity (*i.e*., dFWHM/d*p*), (h) *x*-coordinate, (i) *y*-coordinate and (j) *S_R_* values of the MgGa_2_O_4_:Mn^2+^ phosphor. (k) Pressure-dependent emission spectra of the MgGa_2_O_4_:Mn^2+^ phosphor.

**References**

1. G. Blasse, Energy transfer in oxidic phosphors, Phys. Lett. A 1986, 28 444-445.

2. D. L. Dexter, A theory of sensitized luminescence in solids, J. Chem. Phys. 1953, 21, 836-850.
